# Georgia Medical Association

**Published:** 1882-04-20

**Authors:** 


GEORGIA MEDICAL ASSOCIATION.—As we go to press before the meeting of the Association, we cannot notice its proceedings in the present issue, but will do so in our next. At this writing the indications give promise of a large and interesting meeting. The meeting of the Association is appointed for the 19th inst., in this city.

